# Eleven quick tips for Biomedical Federated Learning

**DOI:** 10.1371/journal.pcbi.1014530

**Published:** 2026-08-21

**Authors:** Kyle Ellrott, Venkat S. Malladi, Jean-Christophe Bélisle-Pipon, Emek Demir, Yael Bensoussan, Serghei Mangul, Alex A. T. Bui, Paul C. Boutros

**Affiliations:** 1 Department of Biomedical Engineering, Oregon Health & Sciences University, Portland, United States of America; 2 Division of Oncological Sciences, Knight Cancer Institute, Portland, United States of America; 3 Health Futures, Microsoft Research, Redmond, Washington, United States of America; 4 Healthcare and Life Sciences, TTEC Digital, Austin, Texas, United States of America; 5 Faculty of Health Sciences, Simon Fraser University, Vancouver, British Columbia, Canada; 6 Department of Molecular and Medical Genetics, Oregon Health & Sciences University, Portland, United States of America; 7 Department of Otolaryngology-Head &Neck Surgery, University of South Florida, Tampa, United States of America; 8 Sage Bionetworks, Seattle, Washington, United States of America; 9 Department of Biological and Morphofunctional Sciences, College of Medicine and Biological Sciences, Stefan cel Mare University of Suceava, Suceava, Romania; 10 Department of Computers, Informatics, and Microelectronics, Technical University of Moldova, Chisinau, Moldova; 11 Department of Radiological Sciences, University of California, Los Angeles, Los Angeles, California, United States of America; 12 Institute for Precision Health, University of California, Los Angeles, Los Angeles, California, United States of America; 13 Sanford Burnham Prebys Medical Discovery Institute, La Jolla, California, United States of America; SIB: Swiss Institute of Bioinformatics, SWITZERLAND

## Abstract

Modern statistical and machine learning techniques are effective at describing, testing hypotheses and making predictions from complex data. This effectiveness is strongly influenced by the volume and heterogeneity of available data. In many fields, including much of biomedicine, large centralized datasets are not available because of cost, privacy, regulatory or other restrictions. In these cases, smaller datasets are distributed across a large number of independent sites. Medical record data is a classic example of this challenge: the total number of patients may be large, but their records are distributed across many health systems and cannot easily be centralized. Federated learning (FL) is a machine learning paradigm that enables training and validation of a shared model in settings of decentralized data. FL can improve model accuracy and generalizability by increasing sample size, but has trade-offs ranging from operational complexity to data-privacy risks to the potential to introduce unexpected imbalances in model accuracy. We outline ten tips for successfully and sustainably implementing FL for Biomedical applications, ensuring both ethical data governance and improved model performance in sensitive domains.

## Introduction

Modern machine learning (ML) methods can leverage extremely large datasets to build high-performing, generalizable models. It is often the case that no single data source provides sufficient training information to optimize model accuracy, eliminate bias, and establish generalizability. Centralizing and pooling data from multiple sources is a classic solution to this problem, and is sometimes called a “mega-analysis.” Still, centralizing data can be challenging for several reasons. First, it centralizes the costs of computing and storage. Second, it can require time-consuming execution of a large number of data sharing and collaboration agreements to manage a range of strategic and regulatory issues. Third, it creates a single point of failure for security issues. For these and other reasons, training ML models on pooled datasets is often infeasible.

The ability to aggregate hidden datasets enables biomedical research. Healthcare systems, research centers, and controlled-access government-funded research datasets all sit in myriad computational facilities and all have different complications around accessing them. The scale of data required for learning complex biomedical systems is extreme, but the individual datasets are tiny in comparison. In the United States (US), there were over 2 million new cancer cases in 2025 [1], but they are spread over all 50 states, so that even the state with the most cases, California, only sees 10% of those patients. For rare diseases, or complex datasets that require a large number of samples to understand, no single hospital may see enough patients to collect a cohort sufficient for building accurate models.

Federated learning (FL) is a key component to address this problem. FL enables the training of a shared model on decentralized, independently maintained datasets. Similar to a meta-analysis, FL enables artificial intelligence (AI) at scales that would be otherwise not feasible [2–4]. The most common FL implementations involve many sites/devices, each of which trains a model on its own local data. These local models are then combined at a central server and aggregated into a global model. Iteration and convergence strategies can then be applied, along with cross-device validation. In many ways, these types of FL are a form of ensemble learning, where models are trained on device-specific samples of the data. As a result of its decentralized nature, federated learning can preserve the privacy of training data. Training outputs, such as model weights, are shared while the data, such as patient records, are kept private. As a result, it is possible to train large-scale models while respecting governance and legal frameworks like the Health Insurance Portability Accountability Act (HIPAA). Beyond the immediate benefits of privacy preservation, federated computing can also provide a framework for accumulating data and computational resources required to build foundation models [5, 6]. However, FL is not a privacy panacea [7]. It changes the locus of risk and accountability rather than eliminating it [4]. In biomedical settings, FL should be framed as a *sociotechnical* intervention governed by necessity and proportionality: use FL when it is the least intrusive means to achieve a legitimate scientific aim, and pair it with concrete governance duties (joint controllership or controller-processor clarity, auditable access, and public-facing documentation).

FL has been successfully applied in a number of biomedical contexts, including COVID-19 outcome prediction [8], chest x-ray classification [9], and medical image segmentation [10]. Given the widespread interest in clinical data analysis, the appeal of federated learning has been increasing. At the same time, its practical initiation often presents significant complexities, a propensity for errors, and technical difficulties [11].

In this paper, we have collected 11 tips to help build a FL network. One of the easiest ways to guide yourself through each of these tips is the table of questions that should be asked (Table 1). These tips are not about the technical aspects of a FL network, but rather the social issues that need to be navigated. Following these tips should address common issues and maximize the short- and long-term success of the new FL initiative.

**Table 1:** Questions to ask.

Tip 1: Assess the Benefits of FL Approaches	• Will federated learning help build a more accurate model? • Is there extreme class imbalance in the datasets, and can it be addressed?
Tip 2: Secure Early Engagement from IT & Security Teams	• Does data in the training set fall under HIPAA or other protection? • What systems will be used for computation? • What security audits will need to be done to be in compliance with institutional standards? • Who will be responsible for which aspects of security and compliance? • Is there a level of security that all participant sites agree to that meets a common acceptable baseline?
Tip 3: Assess Risks to Privacy and Fairness	• What sensitive information could be inferred from model updates or outputs? • Are there disparities in model performance across institutions? • What protections are in place/required and are they appropriate?
Tip 4: Establish Project Governance	• Who are the data owners and what rules are already established regarding its usage? • How will issues in governance be reported, including data leakage? • How will requests to remove data or add sites be handled? • How will intellectual property (IP) from the model be managed?
Tip 5: Define the First Version of Federation Platform	• Which platform will be used? • How will billing for compute/storage usage be handled?
Tip 6: Start With an Example Dataset At Two Sites	• Is there an existing synthetic or open access dataset that can be used for prototyping?
Tip 7: Stress Test with Models of Escalating Complexity	• What is the largest model our current deployment could accommodate?
Tip 8: Pressure Test Security & Governance	• If a bad actor was able to send models to the system, is there a way they could game the system to retrieve identifiable data?
Tip 9: Lock Data & Model Standards	• What would the version 1.0 of the system and datasets look like? What would version 2.0 be?
Tip 10: Perform Descriptive Analyses & Verify Offline	• Do the results match expectations? • Do the stakeholders perceive any gain from the effort required for setting up the FL framework?
Tip 11: Ensure Proper Documentation	• If a new individual had to use or maintain the system, how would they learn?

## Tip 1: Assess the benefits of FL approaches

The first question any FL project must answer is whether it will improve model performance. It is recognized that the performance of learning models, particularly when addressing complex problems, can improve with the incorporation of extensive datasets. This is particularly true for highly parameterized learners, like deep learning (DL) models. The data security and privacy benefits of FL can encourage entities to make available samples that would otherwise be inaccessible for regulatory, ethical, or competitiveness reasons. Acquiring and harmonizing large datasets is often costly and time-consuming. FL offers a way to scale data collection, distributing the efforts of hosting, cleaning, and maintaining a robust compute environment.

But FL is not the optimal solution under all scenarios. Harmonized, centralized repositories enjoy a number of advantages. For example, distributed data can lock in systematic differences in data characteristics across sites; this is a version of “batch effects.” These differences can be subtle and difficult to quantify in a distributed framework. Similarly, aggregating multiple “weak” learners can lead to poorer handling of rare cases than models fit on full datasets. In training of DL methods, samples are typically shuffled to ensure optimization occurs on a diverse set of data. The site-specific structure of data in FL can limit data shuffling, creating *order bias*, where the optimization process struggles against a moving objective due to local batch effects and a lack of global data diversity [12]. In ML and reinforcement learning (RL) analyses, the “long tail” effect can occur when there are a large number of rare cases, leading to a marginal improvement per sample as the dataset grows. If an event is only seen once or twice at each site, there is no way to aggregate these solitary events across different sites into a larger collective set to mitigate class imbalance. Methods for accounting for long tails can partially mitigate these issues, but are not commonly applied [13]. The limitations of FL can counterbalance the benefits of increasing sample size such that overall model performance may not increase even as federated sites are added.

Thus, the first step in any FL project is to determine whether it will improve the performance of models. A thorough evaluation of this process necessitates a rigorous cost-benefit analysis, weighing the prospective advantages of an augmented sample size against the potential magnitude of confounding batch effects.

## Tip 2: Secure early engagement from IT and security teams

Once the potential benefits of FL are established, the subsequent phase involves close collaboration with both information technology (IT) and compliance departments. Within a complex health IT ecosystem, the deployment of an FL platform often requires the engagement of various IT teams, including those specializing in network and compute infrastructure, devOps, security, and regulatory compliance, among others. The restrictions and complexities are compounded in environments holding protected data, like identifiable or limited/sensitive datasets [14]. Most FL setups involve multiple organizations, each with their own IT ecosystem, cultures, and policies. Bringing these groups together at the outset, with clear support from IT leadership and in many cases an executive sponsor for the FL project, is necessary for success.

One example of this complexity is the inter-institutional communication required in a federated learning system. A common workflow involves an external-facing endpoint that receives requests, allocates compute and data resources, runs workflows, and finally transmits results to another endpoint at another institute. This often necessitates executing arbitrary or weakly-defined training and analysis code, as well as communicating through specific ports and to specific IP addresses.

For many organizations, such a workflow requires exceptions to security rules and buy-in that typically goes far beyond a simple approval. The resultant expansion of an organization’s attack surface entails acceptance and active participation from appropriate security stakeholders to recognize the mission-driven benefits and proactively minimize risks. IT and security teams need to conduct thorough security reviews, document risk factors, and establish mitigation strategies on an ongoing basis. They are engaged in defining and documenting ingress and egress ports, implementing robust monitoring and logging mechanisms. Key considerations include:

- Data encryption: Ensuring that data is encrypted both in transit and at rest across all participating sites.
- Authentication and authorization: Implementing secure authentication and authorization protocols to control access to federated learning resources.
- Audit trails and review: Establishing comprehensive audit trails to track data access and system activity.
- Vulnerability scanning: Regularly scanning federated learning infrastructure for vulnerabilities and applying necessary patches.
- Cost of computational time: AI/ML capable machines, namely those with large memory and GPU capabilities can be much more expensive to request, especially in a cloud infrastructure. Without proper moderation, a large number of scheduled jobs could become a form of Denial of Service (DOS) or Economic Denial of Sustainability (EDoS) driving project costs with excessive cloud fees.

With all of these considerations, start a dialog with IT and security teams as soon as possible. These conversations can take the longest and are also the most crucial, so starting early will save time down the line.

## Tip 3: Assess risks to privacy and fairness

Privacy is a primary driver for adopting FL in biomedical research, so evaluating privacy risks must sit at the heart of every development plan. Before any code is written, project leaders should enumerate the types of information that could be inferred from model updates. These include concepts such as allele frequencies, rare-variant patterns, or clinical outcomes. A project must also assess the likelihood of re-identification (PMID 39081567). The algorithm’s structure and the granularity of the underlying data determine the exposure surface. For example, summary-statistics algorithms such as GWAS meta-analysis can leak individual counts, but this risk is mitigated when sites prune low-frequency samples [15]. Generative models, including GANs and diffusion models, pose a threat as they may unintentionally memorize and reproduce patient-specific records [16]. Algorithmic frameworks such as differential privacy [17] or secure multiparty computation [18] can provide additional layers of protection, but these solutions may be specific to the task or problem domain and the implications must be considered as part of the problem analysis.

Additionally, as projects and cohorts grow, governance should draft plans for fairness and address the possibilities of data/subject withdrawal and unlearning. Security tests should be paired with fairness and reversibility tests. Another strategy is to build a federated fairness audit that: (1) computes per-site and per-subgroup performance with confidence intervals; (2) flags contribution-benefit gaps (sites contributing rare cases but receiving poor local performance); and (3) halts aggregation when fairness deltas exceed pre-set bounds. This report can be published with the audit summaries to all nodes. To ensure compliance with potential issues with institutional review boards (IRBs) there should be a plan to operationalize federated unlearning: this would involve defining triggers (participant withdrawal, consent revocation, data error), select a method (client-level or update-level unlearning), and test rollback to pre-event checkpoints. Finally, calibrate differential privacy: document the privacy budget and expected utility loss at node-level so privacy protections do not disproportionately degrade models for under-resourced sites.

## Tip 4: Establish project governance

Although FL reduces the risk of inadvertent data sharing, participation still requires clear agreements and proper scientific governance. Despite the lowered risk of inadvertent sharing of controlled data, participation in an FL network, even one comprising just two sites, requires several issues to be agreed upon up-front. Typically, legal agreements are needed to allow ready transfer of code, model structures, model weights, and even example datasets. These agreements should include shared understanding about intellectual property rights, expectations about intended use of the service, and other issues. For instance, a key consideration is whether research focused on identifying individuals within a federated learning network would be deemed appropriate and acceptable to all parties. If a potential breach or privacy violation is identified, how should that be reported? Are there limitations on the expectations of how intensively sites employ one another’s compute and network resources? How should notifications about changes to a dataset be made (*e.g.,* withdrawal of consent from a subset of study participants)? What about changes to the underlying FL code? Is there a process to expand the FL network to additional sites? If a site elects to withdraw, what rights and obligations survive their departure? A clear governance framework is needed to address these questions at the outset.

Start with creating a written Federation Agreement that covers: (1) role allocation under data-protection law (joint controllership vs. controller-processor); (2) a registered/controlled/open access tiering for code, metadata, and model artifacts; (3) a Data Access Committee Office (DACO) style workflow with turnaround service level agreement; and (4) explicit conditions for consent withdrawal, institutional withdrawal, and model incident reporting. Plan to document a breach/poisoning response plan, including model rollback and federated unlearning triggers. Academic and hospital IRBs and General Councils may still be quite unfamiliar with this new technology, so bringing supporting documentation to explain the technology and ethical risks to the governing entities of the institution will be crucial to proper implementation. In cases of studies involving prospective biomedical data collection including a consent process, the FL technology should be explained to participants using lay language describing the risks and benefits of this technology within the informed consent form (ICF) to gain their trust.

To manage biomedical data, one should treat governance as the core deliverable [19].

## Tip 5: Define the first version of the federation platform

Choosing an FL platform and compute resources are a strategic decision with long-term implications for security, compliance, interoperability, and cost. The landscape of FL platforms is currently fragmented, with numerous independent solutions available and under development (Table 2). This lack of standardization presents a significant challenge to interoperability and model sharing. The initial and often foundational decision in establishing a federated learning network entails a strategic selection of a platform and its associated compute resources. This decision is far more than a technical implementation: it has long-term implications for security, compliance, interoperability, and financial sustainability.

**Table 2:** Federated learning frameworks review.

Name	Framework	Developer	Homepage
OpenFL	Tensorflow, PyTorch	Intel	https://openfl.readthedocs.io
TensorFlow Federated	TensorFlow	Google	https://www.tensorflow.org/federated
APPFL	PyTorch	DoE	https://appfl.readthedocs.io/
NVFlare	Tensorflow, PyTorch	NVidia	https://nvflare.readthedocs.io
Flower	Tensorflow, PyTorch	Startup	https://flower.dev
FATE		Tencent	https://fate.fedai.org/

Compute resources also need to be specified proactively. Elements such as memory capacity, CPU manufacturer/model and speed, and storage capabilities are all critical factors. GPU availability is perhaps the most critical aspect of resource management for contemporary modeling approaches. The GPU memory capacity may have significant effects on the training performance for some classes of problems, with low-memory GPUs being unable to run larger models.

Decisions about resource allocation are critical, not only for feasibility but because of the fiscal implications of a federated learning topology. External groups are sending requests to the data host, and those requests are carried out using the host computing facilities. This means the host will be billed for any costs incurred as part of the computation. These billing issues may be more discrete for on-premises facilities, but they manifest as electric bills and system queue availability. In cloud-based systems, this could translate into larger billed time on some of the most costly virtual machines (VMs) offered by the cloud provider. It may be prudent to identify usage limits, or develop agreements around reciprocal usage or charge-back policies. If not managed correctly, a federated endpoint could become a monetary “denial of service” endpoint allowing outside entities to impose unwanted financial costs on the host.

Defining the system requirements, both for software and hardware, as part of the federated platform specification, will enable a better understanding if the platform will be ready for the scale of the proposed learning problem.

## Tip 6: Start with an example dataset at two sites

While working to establish and verify IT protocols and governance, a project can create momentum by developing test scenarios around safe example datasets that involve minimally encumbered data (*i.e.,* simulated federated learning). Public data is ideal for initial test scenarios, but any dataset that has minimal security and legal risk, and allows inspection of a unified dataset from both sides can be useful in this stage.

In some cases, development of this example dataset can be difficult, especially when scale is valued. In such cases, synthetic datasets may be ideal. For example, to test electronic health records (EHRs), there is Synthea [20]; SyntheVAEiser [21] for transcriptomic data; and other methods for H7E image generation [22]. Other reviews have been written on the topic [23] and can help guide researchers to tools that can be used to create new datasets for testing.

It is also useful if the example dataset contains actual signals and supports multiple question types. Statistical tests should be built into the example dataset, such as batch-connected alterations to the data. These can include shifts in the population distributions, introducing missingness, and embedding noise into different variables at different sites and including “N/A” (not available) or “NaN” (not a number) values in only one of the datasets.

By having more than one institution work on this problem, it provides an opportunity to start validating data standards and testing ideas around the problem space. But this phase should also be treated as a “warm up,” including a large number of institutions early on where the datasets are meaningful. This aspect requires both an understanding of why the data is acquired, and how it is curated. Endeavors representative of the University of California Data Health Warehouse and the NIH Bridge2AI Data Generation Projects [24] are notable here, appreciating how specific efforts are able to collect data within specific scopes.

This rule can also be extended to keep the FL system as simple as possible. If a certain aspect is not going to be needed by at least two sites, it can be scheduled for a later time. This principle keeps feature creep under control, which is especially critical in the current rapidly changing environment.

## Tip 7: Stress test with models of escalating complexity

Synthetic datasets are useful for early testing but are limited in scientific utility. Therefore, after the initial validation, the pipeline should be stressed with progressively more complex data and models. In a synthetic dataset, any signal needs to be designed and baked into the data, so without significant investment in research and development in embedding realistic signals ML models trained on synthetic data will be unable to detect and learn any useful patterns. Developing complex and realistic signals in a synthetic dataset can be an increasing time sink, and the project team should determine the amount of time that should be invested in improving more complex synthetic datasets.

As data and model size and complexity increase, many of the nuanced technical challenges inherent in machine learning become more significant. To facilitate preliminary analysis and identify such potential issues early on, it is prudent to begin with simpler models. Starting with easily interpretable models like basic linear models and decision trees, which allow for a faster iteration cycle and helps pinpoint problems with the platform and data generation, cleaning, and formatting stages. As initial tests are successful, move to slightly more complex models like random forests and single-layer neural networks, before progressing to large-scale DL. While extremely large models may ultimately be needed to achieve top-tier performance, they also present increased risk of instability, heightened sensitivity to parameter tuning, and potential for unexpected failures. Once the foundational testing loop has been thoroughly validated with simpler models, the project can then progressively introduce models of increasing complexity.

Modern AI systems, particularly in federated learning environments, face significant challenges with robustness and stability. This is largely due to their distributed and sectioned nature, where artifacts like data, models, and software are updated independently. To address this, it’s crucial to implement a continuous integration (CI) testing philosophy. All tests, once developed, should be isolated to reduce compute costs and integrated into a testing suite that is run frequently. This approach ensures all stakeholders have transparent access to the results, allowing for proactive identification and resolution of issues.

## Tip 8: Pressure test security and governance

Even as the example dataset is identified and consideration is given to the analyses to be supported, it is critical to also recognize how assumptions made around security and governance may fail. While many existing platforms incorporate security measures, these are often designed to defend against external, out-of-band attacks. A more rigorous evaluation is required, encompassing “white hat” intrusion testing and vulnerability assessments to identify and address potential weaknesses before broader researcher access is granted.

Federated learning presents unique security challenges. A critical concern is the potential for protected information to be inadvertently leaked within model weights [25, 26]. Efforts should be made to reconstruct or infer protected fields as a form of ‘white hat’ hacking experiment [27, 28]. Surveys on the strategies for securing federated learning systems have been performed [28] and outline strategies to minimize the risks of sensitive data leakage.

The system must be safeguarded against “model poisoning” [29], where malicious or even unintentionally biased participants contribute optimized weights that skew the aggregate model. This results in models that lose confidence on calls, become less generalizable, and have worse overall performance. Batch effects result from genuine data incongruities or unforeseen batch effects, highlighting the importance of robust validation and anomaly detection mechanisms. While not the result of malicious intent, batch effects can still have similar effects as model poisoning. Similar strategies of monitoring overall performance changes and correlating with dataset providers and help identify the origins of spurious training intervals. By way of illustration, different types of batch effects can become apparent as the real datasets become available per site, including differences in data distributions (*e.g.,* Site X has 65% of cases, Site Y has 25%, etc. each with different case mixes) that propagate imbalanced model learning due to underlying site-specific biases; questions of fairness in FL thus arise in different ways that should be identified earlier in the process [30–34].

Security and governance testing should not be treated as a one-time event at the beginning of the project. These elements should be revisited at common intervals, including periodic security inspections, project review and software updates to ensure security-related patches as incorporated into the federation platform. Logging from all requested jobs can be reviewed to ensure that usage patterns follow expectations laid out by project descriptions.

## Tip 9: Lock data and model standards

Software development is iterative, but for FL it is essential to lock data and model standards before a large-scale learning cycle begins. Developing a versioning system before starting a cycle of federated learning is incredibly important for both debugging and provenance. Options include using semantic versioning [35], tags generated from checksumming file and code manifests (*e.g.*, Secure Hash Algorithm), or a date-based system. When a model is trained, the model code and dataset version numbers should be recorded as part of the training process’s metadata to enable reproducibility in the future. If external pre-trained models are used, they should also be version controlled. These efforts will help to identify issues of changing model performance over time, and help identify if updates to datasets have had unintended side effects.

## Tip 10: Perform descriptive analyses and verify offline

As the project progresses from freely accessible example datasets to controlled access, real-world datasets, visibility into training results will inevitably decrease. This transition will expose true batch effects and data biases not apparent with the initial testing data. Critically, access control rules may now restrict individual access to both component datasets, further complicating debugging and result evaluation. To proactively address these challenges, project teams must establish robust descriptive analysis methods and offline verification processes. These methods should prioritize anonymized data exploration and summarization, allowing teams to identify and investigate trends in the training data without compromising the privacy and security of the underlying samples. Specifically, teams should explore techniques for generating aggregate statistics, visualizations, and summary reports that can be shared and discussed collaboratively. Additionally, the experience of the team members of each site should be collected periodically: (1) to understand “pain points” from the effort, addressing the challenges in deployment; and (2) to evaluate the impact of the learned model given their site’s input into helping to create it (and thus ongoing participation in the endeavor). Not only are such insights important for future FL-based projects but also to validate the cost and utility of the shared effort.

## Tip 11: Ensure proper documentation

A federated learning framework is a consortium: no one person can drive the entirety of the project. The “bus factor,” a term from software engineering, quantifies the systemic risk to a project should key individuals become unavailable, representing the number of people whose loss would cause the project to fail due to critical knowledge or skill gaps. Even for complex projects, this number can be surprisingly low. For FL, loss of skill sets or domain knowledge at any of the sites could remove it from the federation and affect the entire group. One of the most effective ways to combat this systemic risk is to ensure the details of the project can be properly documented. A partial list of items that should be documented includes:

- Rationale for deploying FL
- Compliance and security evaluations
- Governance documents
- Procedure for amending governance documents
- Procedure for adding or removing a data site from the federation
- Procedure for adding or removing an analysis site from the federation
- Code of conduct for sharing of compute resources
- Code and system documentation standards
- Emergency procedures for breaches or other unexpected events
- Security audits
- Backup protocols
- Code samples to scaffold new analyses

Documentation should be living, versioned, and accessible to all participants.

## Conclusions

Most FL literature focuses on its mathematical foundations and algorithmic optimization; practical application of FL requires navigating complex social, administrative and governance landscapes. Similarly, many examples of FL system deployment focus on software; in the real-world, the data cleaning, formatting, and validation are often rate-limiting. The intrinsic value, privacy, size, and legal considerations surrounding healthcare-derived data make FL highly appealing. To avoid bottlenecks that stall or terminate efforts, a holistic approach that proactively jointly addresses technical and non-technical issues is needed.
